# Chemical characterization of *Cassia fistula* polysaccharide (CFP) and its potential application as a prebiotic in synbiotic preparation

**DOI:** 10.1039/d1ra00380a

**Published:** 2021-04-09

**Authors:** Dawood Hosni Dawood, Mohamed Samir Darwish, Asmaa A. El-Awady, Azza H. Mohamed, Ahmed A. Zaki, Mohamed A. Taher

**Affiliations:** Agricultural Chemistry Department, Faculty of Agriculture, Mansoura University Mansoura 35516 Egypt; Dairy Department, Faculty of Agriculture, Mansoura University Mansoura 35516 Egypt msamir@mans.edu.eg +20 1005838367; University of Florida, IFAS, Citrus Research & Education Center 700 Experiment Station Road Lake Alfred FL 33850 USA; Pharmacognosy Department, Faculty of Pharmacy, Mansoura University Mansoura 35516 Egypt

## Abstract

Prebiotics are non-digestible food ingredients that are selectively fermented by probiotics. The aim of this study was to investigate the chemical properties of a polysaccharide extracted from *Cassia fistula* mature fruit pulp and to evaluate its effects on probiotic strains: *L. casei*, *L. rhamnosus*, *E. coli* Nissle 1917 (EcN), and *E. faecalis*. These strains were compared for their growth behavior in culture media supplemented with different *Cassia fistula* polysaccharide (CFP) concentrations. The molecular weight of CFP was approximately 8.707 × 10^5^ Da. The recovered polysaccharide contained a low percentage of crude protein (4.4%). Aspartic acid, glutamic acid, and proline were the most abundant amino acids. Glucose and mannose were the predominant sugars followed by arabinose and rhamnose. *L. casei* grew faster at high CFP concentrations (2%) compared with the lower concentrations of CFP. The highest values for the prebiotic index and prebiotic activity score were observed for *L. casei* treated with 2% CFP, and it may be considered a prebiotic due to its high resistance against α-amylase and acidic conditions. CFP provides two ways to adjust nitric oxide (NO) synthesis in macrophages. Finally, the use of 1.5 and 2% CFP for cultured milk production significantly shortened the fermentation period from 210 min to 180 min and 150 min, respectively.

## Abstract 2

CFP may be a prebiotic due to its high resistance against α-amylase and acid. CFP provides two ways to adjust nitric oxide synthesis in macrophages. We show the use of CFP in the production of synbiotic yogurt with favorable physical and chemical characteristics.

## Graphical abstract

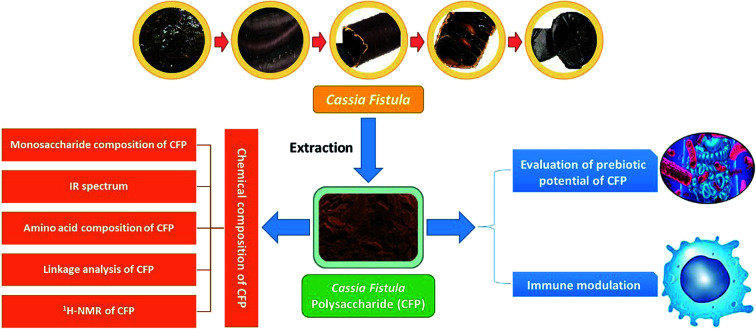

## 1. Introduction

*Cassia fistula* is a medium-sized, fast-growing, deciduous tree native to Srilanka, India, and the Amazon region. It is extensively cultivated worldwide as an ornamental tree for its beautiful yellow flowers. In Brazil, the aerial parts of *C. fistula* are used to treat inflammation and the seeds are used as a laxative. Overall, phytochemical studies of the Cassia genus have shown that it is commonly used in traditional medicine as a purgative, diuretic, antiseptic, and an antioxidant to treat ulcers, jaundice, and various skin diseases including leprosy.^1,2^ Previous phytochemical studies of *C. fistula* extracts revealed the presence of coumarins, alkaloids, glycosides anthraquinones, and phenolic constituents.^3^ Additionally, the ripe fruit pulp contains a significant amount of anthraquinones in the form of rhein as well as soluble sugars, volatile oils, and resins.^4^ Purified rhamnetin 3-O-gentiobioside (from roots), sennosides A & B (from leaves), fistulin and kaempferol (from flowers), isoflavone biochanin A (from fruits), and three new compounds designated cassioates D, E, and F (from the whole plant) have been previously isolated from *C. fistula*.^1,5,6^ The aforementioned secondary metabolites are well-recognized as exhibiting various biological activities. For example, *in vitro* antioxidant activity of hydroalcoholic seed extracts of *Cassia fistula* has been reported.^7^ Amentoflavone isolated from *C. fistula* leaves exhibited cytotoxicity against HepG2 liver carcinoma cells and had antioxidant activity.^8^ Neuroprotective and antioxidant activities of an aqueous methanolic extract from *Cassia fistula* leaves using a *Caenorhabditis elegans* model has been observed.^9^ Overall, the recent studies on *C. fistula* have focused on the ripe phase of the fruit pulp and diverse biological properties such as hypolipidemic, anticancer, antioxidant and antibacterial abilities have been stated.^10–12^

Polysaccharides are known as vital biological macromolecules for all living organisms, which are structurally composed of homo or hetero monosaccharides connected with glycosidic bonds. In *C. fistula*, different methods of extraction have been described to extract the crude polysaccharides content including extraction with boiling water, boiling ethanol, chloroform water IP or 1% NaCl solution.^13^ Polysaccharides were then precipitated by treating the extraction solution in several volumes of ethanol. Preliminary chemical investigation of purified *C. fistula* seed and pulp polymers indicated the presence of proteins in the seeds and pulp polymers showed carbohydrates and mainly hexose sugars.^13^*Cassia fistula* seed polymers were sparingly soluble in water, and pulp polysaccharides were entirely soluble in water. Both seed and pulp polymers were insoluble in ethanol and chloroform.^13^ The chemical compositions of crude gum of *Cassia fistula* seeds was previously determined by Huanbutta and Sittikijyothin.^14^ The percentages of moisture, protein, fat, ash and polysaccharides were 4.29, 1.03, 10.04, 0.09 and 88.84, respectively. The backbone of *Cassia fistula* seed polysaccharide is a linear chain of β 1,4-linked mannose residues to which galactose residues are 1,6-linked at mannose, forming short side-branches.^15^ The seeds mucilage of *C. fistula* has been assessed in tablet formulations as binder.^16^ Carboxymethylated *C. fistula* gum has utilized in aqueous tablet-coating process.^16^

**Fig. 1:**
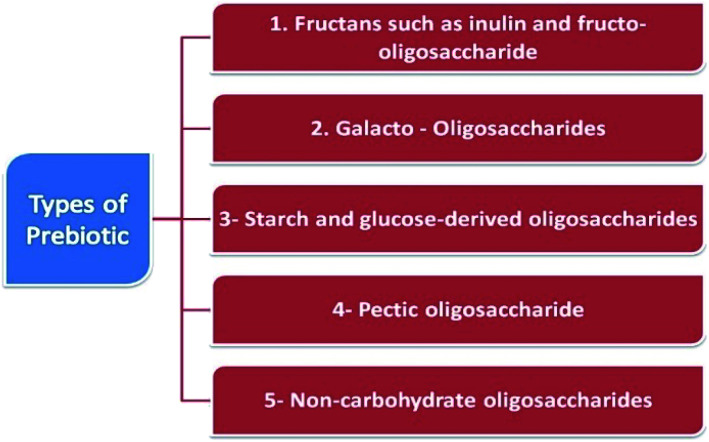
Classification of prebiotics.

The global functional foods market has witnessed growth in the last few years due to increasing healthy food awareness of consumers toward functional foods which are claimed to improve consumer health. Regardless other food components, polysaccharides are among those components that have acquired clear attentiveness recently, for their positive biological effects.^17^ The polysaccharides and their derived products have been well assumed as main components in medicine, food, agricultural proposes.^17^ The positive health effects of polysaccharides, such as antimicrobial activities, anti-cardiovascular, immune function and antitumor have been stated.^18^ In the last decade, many natural polysaccharides are extensively used in many industries due to their non-toxicity, biodegradation, biocompatibility, non-immunogenicity,^19^ and prebiotic potential.^20^ The global demand for the use of prebiotics is appeared to be approximately 167 700 tons and to be value of 507 million USD.^21^ Prebiotics are selectively fermented component and non-digestible which allow particular modifications, both in the activity and/or composition in the intestinal microflora which provides benefits upon host health and well-being.^22^ They are intrinsically more resistant to digest by digestive enzymes such as α-amylase in the upper gastrointestinal tract.^20^ There are several prebiotic types (Fig. 1). The mostly of them are a sub set belong to carbohydrate groups and are majority oligosaccharide carbohydrates, but several recent studies reported the evidence proving that some types of prebiotics are not belong to carbohydrates.^23^ Generally, prebiotics serve as food for probiotics. Probiotics are live microorganisms that, when consumed in suitable quantities, confer a consumers' health value by improving or maintaining their balance of intestinal microbial.^20^ The microbial numbers inhabiting the gastrointestinal tract has been recorded to exceed 10^14^, that includes 10 times more than human cell numbers.^24^ Nevertheless, a recent modified estimate has reported that the ration of bacterial: human cells is close to 1 : 1.^25^ The combination of eukarya, archaea and bacteria colonizing the GI tract is characterized the gut microbiota and has co-improved with the human body during millennia to form a mutually and intricate beneficial relationship.^24^ The synbiotics concept is not well developed and demands further research and validation. Roberfroid introduced the prebiotic concept in 1995, and it has become very common since then. However the synbiotic has not been widely used and is a new notion in the improvement of nutraceuticals or functional foods. Synbiotic (“syn” means “together” and “bios” means “life”) is defined as supplement that includes both a probiotic and prebiotic that work together to enhance the microflora in colon.^26^ Prebiotic index and prebiotic activity score considered mathematical equations that reflect the ability of given substrate to activate the growth of a probiotic compared with the other microorganisms. In addition to the effect of given substrate to enhance growth ability of probiotic compared with growth rate of probiotic on a non-prebiotic substrate, such as glucose or other type of sugar used as positive control. Subsequently, polysaccharides have positive values in prebiotic index if the utilization of polysaccharides by probiotic bacteria is higher than the fermentation of glucose or other sugar used as a control. However, polysaccharides have positive values in prebiotic activity score, which indicate the rate of polysaccharides utilization by probiotic bacteria, is greater than other enteric microorganisms.^27^

Several synbiotic relationships between prebiotics and probiotics have been investigated, with a focus on maximizing their valuable effects. Both lactobacilli and bifidobacterial were best recognized to employ prebiotics in GI tract, according to the fact that they include high percentage of glycotransferases and β-fructosidase, respectively that enable them to hydrolyze prebiotic into smaller parts and use it as a substrate over fermentation.^28,29^ Organic acids will be generated by probiotic strains as a main product of fermentation, thus providing an acidic condition in the large intestine that indirectly inhibits the pathogens growth. This mode of action confers prebiotics to significantly play the colonic microbiota composition in gastrointestinal tract,^30^ thus enhancing the host health in return.^31^ These involve improvement of digestion, immune function, fecal elimination, reduce irritable bowel syndrome (IBS) symptoms.^32^ Prebiotic act on the gut associated epithelial cells as well as the systemic and local immune cells, primarily through G-protein coupled receptors (GPCRs) pathways. However, other pathways involving histone deacetylase inflammasome and inhibition pathway have also been used in regulating the effect of immunomodulatory.^33^ The prebiotic may also stimulate a microbiota-independent effect by directly acting on innate immune cells and the gut associated epithelial through the Toll-like receptors. The cumulative influence results in the modulation of innate immunity and maintenance of the epithelial barrier integrity through secretion of anti- and pro-inflammatory cytokines, switches in macrophage function and polarization, dendritic cell and regulatory T-cell differentiation, neutrophil migration and recruitment. Several prebiotics have been well studied, with successful animal and human trials presenting the association between immunity biomarkers and gut mirobes leading to enhancement in human health.^33^ In addition to the technological benefits of the using prebiotic in production of functional fermented milk, whereas its use leads to stimulate starter culture and probiotic strains and consequently incubation period is shortened, thus increasing production efficiency and reducing the cost.^34^

The objectives of the present study were (i) to isolate a new polysaccharide fraction from the fruit pulp of *C. fistula* (CFP), (ii) to characterize its chemical composition using different techniques, (iii) to evaluate the prebiotic potential of CFP (prebiotic index, prebiotic activity score, and CFP digestibility by α-amylase or artificial gastric juice (AGJ)), and (iv) to examine the effects of these components on both nitric oxide (NO) production by macrophage cells and bacterial fermentation of milk during the production of yogurt.

## 2. Results and discussion

### 2.1. The composition of *C. fistula* polysaccharide

The hot-water extract from the dried fruit pulp of *C. fistula* was precipitated with 80% ethanol to give the crude polysaccharides, named CFP which gave 11.25% yield of the dried raw material. The total carbohydrate and reducing sugar content of CFP was 88.70 and 19.8 g/100 g, respectively. Therefore, the calculated value for the total polysaccharide fraction was 68.90 g/100 g (Table 1). The crude fraction of CFP contained a small amount of protein (4.4%). This result was in accordance with the findings obtained by Killedar *et al.*,^12^ who noted the reduced amount of proteins in *C. fistula* fruit pulp polymers while high level was noted in seed polymers. Our results agreed with the preliminary chemical tests of *C. fistula* fruit pulp polymers which confirmed the richness with carbohydrates in the form of hexose sugars.^12^ The chemical composition of CFP in this study was consistent with the results obtained by^35^ for *L. leucocephala* polysaccharide.

Carbohydrate and protein content of CFP, monosaccharide profile, and related amino acids composition

**Table:** 

(A) Composition of CFP fraction (%)	
Parameters	Value
Total carbohydrate	88.70
Total polysaccharide	68.90
Reducing sugars	19.8
Protein	4.4

a *n* (Fuc) : *n* (Rha) : *n* (Ara) : *n* (Gal) : *n* (Glu) : *n* (Man) : *n* (Fru).

**Table:** 

(B) Monosaccharide composition (percentage to *n* fucose)^a^	
Parameters	Value
Fucose	*n* = 1
Rhamnose	4.24
Arabinose	4.65
Galactose	2.71
Glucose	41.96
Mannose	6.15
Fructose	2.25

**Table:** 

(C) Amino acids composition (ng g^−1^)	
Parameters	Value
Aspartic acid	1.581
Threonine	0.596
Glutamic acid	1.015
Serine	0.597
Alanine	0.643
Glycine	0.344
Arginine	0.434
Histidine	0.193
Tyrosine	0.672
Cystine	0.299
Isoleucine	0.351
Lysine	0.519
Valine	0.678
Methionine	0.316
Proline	0.963
Phenylalanine	0.656
Leucine	0.542

### 2.2. Monosaccharide composition of *C. fistula* polysaccharide

**Fig. 2:**
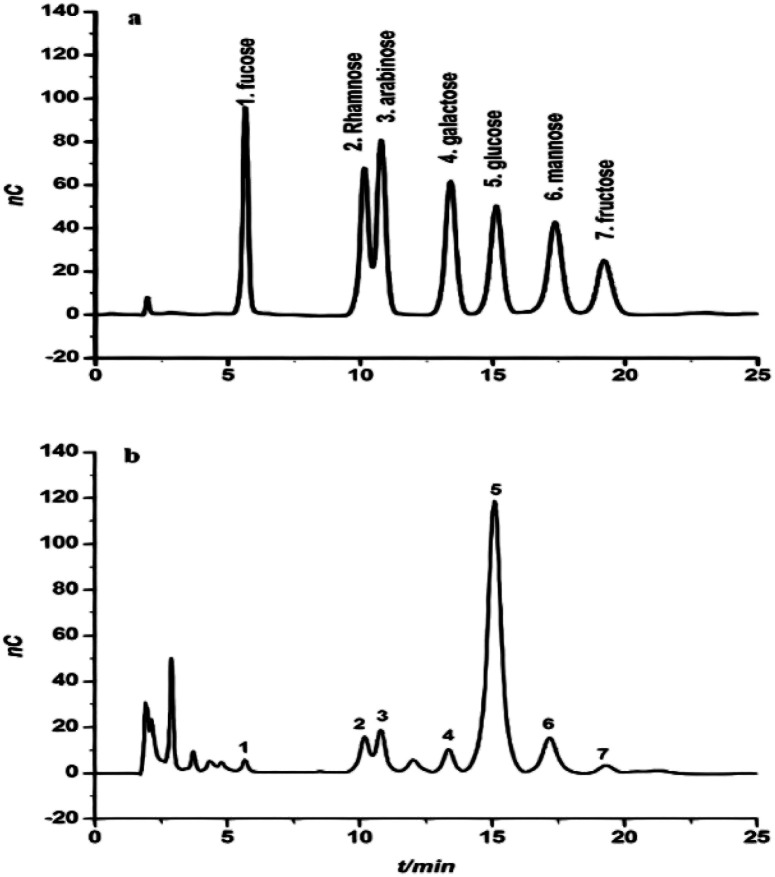
The chromatogram of a standard solution of monosaccharides (a) and the monosaccharide composition of *Cassia fistula* mature fruit pulp (b).

The monosaccharide composition of CFP was determined and validated by hydrolyzing samples of crude polysaccharide in 2 M H_2_SO_4_. A Dionex system (Dx-120) equipped with an electrochemical detector (model ED40) was used with elution in a 15 mM NaOH solution. These conditions were able to separate the monosaccharide fraction of CFP into separate peaks with maximal signal-to-noise ratios (Fig. 2A and B). A similar technique was used to identify the monosaccharide composition of some polysaccharides extracted from the fruiting bodies of palm tree species.^17^ The identification of each peak of the tested sample depended on the retention times of the reference monosaccharides. The relative concentrations of the CFP monosaccharides were determined as mole ratios. Relative to the moles of fucose, the molar ratios of the CFP monosaccharide fraction are presented in Table 1B. CFP composition included seven monosaccharides at different concentrations. Glucose was the predominant monosaccharide with the highest molar ratio. The relative order of the other sugars was as follow: mannose > arabinose > rhamnose > galactose > fructose > fucose. The observed findings were dissimilar with those recently reported by,^17^ who recorded that mannose and galactose were the principal monosaccharides in crude polysaccharide extract isolated from fruiting bodies of *C. humilis* and *C. mitis* trees. Interestingly, the biological activities of glucose rich polysaccharides have been reported.^36^ Structural analysis showed that *Pholiota adiposa* mycelial crude polysaccharide exhibits antitumor activity and glucose was the predominant monosaccharide at a high molar ratio.^36^

### 2.3. Infrared (IR) spectrum

**Fig. 3:**
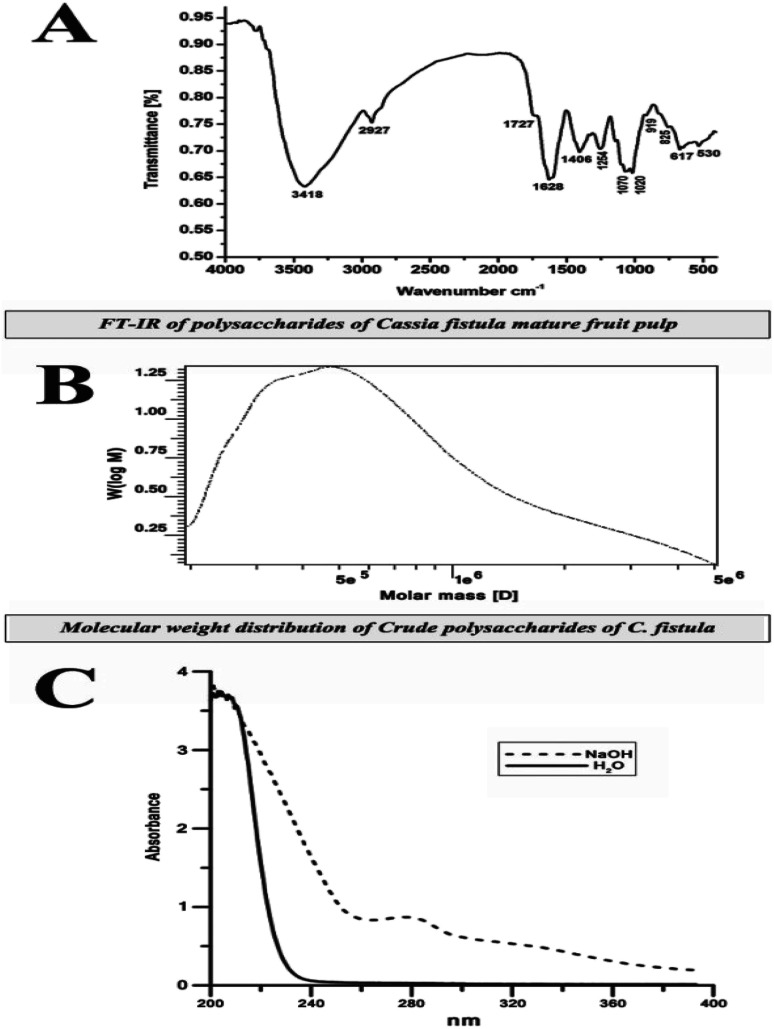
Characterization of CFP (A) FT-IR spectra (B) molecular weight using high performance gel permeation chromatography (c) determination of glycan–peptide linkage before and after treatment with NaOH.

Fig. 3A shows the IR spectrum of CFP in the range of 4000–500 cm^−1^. A broad-stretching typical peak at 3418 cm^−1^ was evident for the hydroxyl (OH) groups in the basic sugar residues. The slight band at around 2927 cm^−1^ was correlated to the elongating vibration of C–H in the sugar structure.^37^ Polysaccharide absorbance values in the series 1000 to 1200 cm^−1^ for C–O–C and C–O–H link band locations have been described.^38^ The absolute stronger absorption peak at 1628 cm^−1^ for the N–H bending vibration may be related to the protein portion of the polysaccharide. The absorbance at 1406 cm^−1^ can be allocated to the methyl C–H wagging vibration.^36^ Moreover, the small absorption bands at 919 cm^−1^ in IR spectra may be associated with β-glycosidic links between the sugar units.^39^ In addition, the minor absorption bands at approximately 919 cm^−1^ in the spectra may be associated with β-glycosidic links between the sugar units.^40^ The bands ranging from 1020 to 1100 cm^−1^ and from 530 to 617 cm^−1^ were assigned to skeletal modes of pyranose rings.^41^ Consequently, we proposed that they are related to β-type heteropolysaccharides with a pyran moiety.

### 2.4. Molecular weight of CFP

High performance gel permeation chromatography (HPGPC) has been used as an effective technique for the estimation of polysaccharide MW.^42^ In this study, CFP exhibited a single, broad peak indicating the homogeneous distribution of the structure (Fig. 3B). The MW of the CFP was calculated to be approximately 8.707 × 10^5^ Da. The purification of plant polysaccharides with such a high MW value has been previously reported.^43^ There have been no previous reports describing the MW of *Cassia fistula* polysaccharides.

### 2.5. Amino acid composition of CFP

Table 1C lists 17 free amino acids in the polypeptide portion of the CFP structure. The predominant amino acids in CFP were aspartic acid, glutamic acid, and proline at concentrations of 1.581, 1.015 and 0.963 ng g^−1^, respectively. Cysteine (0.299 ng g^−1^) and histidine (0.193 ng g^−1^) were present in lower amounts. As shown in Table 1C, the presence of considerable amounts of serine (0.597 ng g^−1^) and threonine (0.596 ng g^−1^) are in the accordance with the O-glycosidic linkage in CFP.^44^ There is no available literature regarding the amino acid content of *Cassia fistula* polysaccharide. This is the first report describing the amino acid content of CFP.

### 2.6. Linkage analysis of CFP

Glycan–peptide linkages in glycoprotein structures can be classified into two types depending on their stability to alkali treatment: N-glycosidic and O-glycosidic linkages.^45^ β-Elimination is the reaction which determines whether the linkage form of the glycoprotein is O-glycosidic. In the presence of NaOH, threonine and serine in the CFP O-glycosidic linkage convert to α-aminocrotonic acid and α-aminoacrylic acid, which results in the absorption at 280 nm. Fig. 3C shows that the absorption at 280 nm increased, indicating that the linkage type of the glycoprotein was O-glycosidic. A similar trend was also detected in the glycoproteins extracted from the fruiting bodies of some palm trees.^17^

### 2.7. ^1^H-NMR of the *C. fistula* polysaccharide

**Fig. 4:**
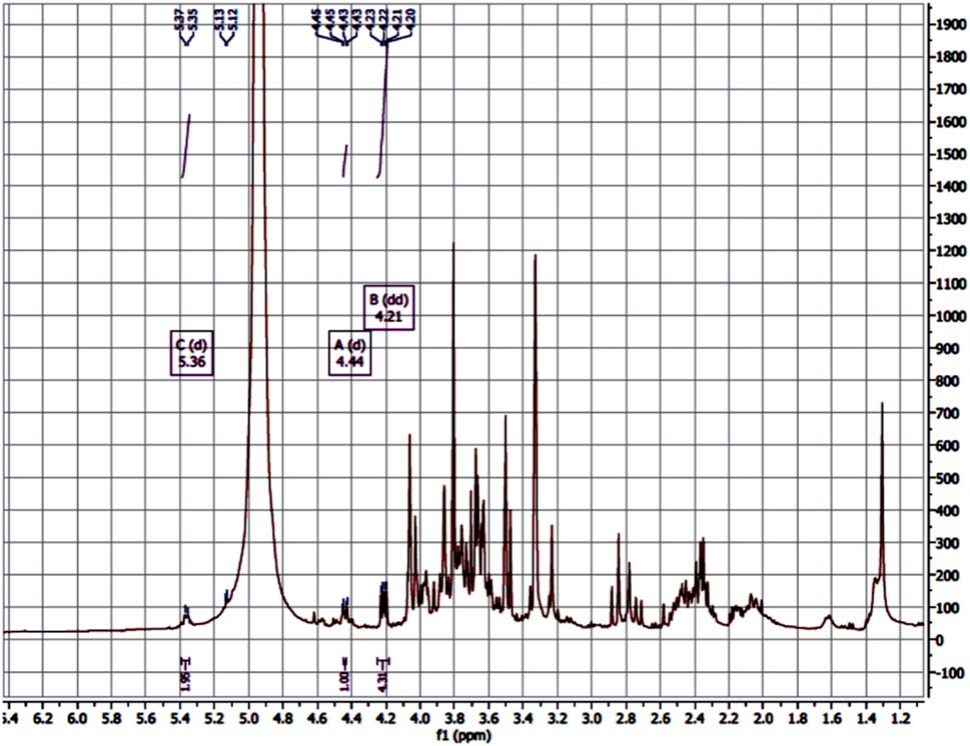
^1^H-NMR of the *C. fistula* polysaccharide.

The ion chromatography analysis of the polysaccharide revealed that the monosaccharide (glucose) is the predominant one followed by mannose. The glucose backbone linkage of CFP could be detected by the ^1^H NMR and investigate the anomeric proton of the glucose units. The down field shift of the anomeric protons made them isolated in the crowded spectrum and their assignment is possible. The anomeric protons at *δ* 4.44 and 5.36 are 280 doublet with *J* = 7.7, indicated the β-d-glucose (Fig. 4).^46^ The polysaccharide extracted from the endosperm of *Cassia fistula* seed and revealed the mannose backbone consists of β-d mannose units linked together through β-1→4 glycosidic linkage. Therefore, the β-1→4-glycosidic linkage of glucose backbone of CFP is similarly proposed.^15^

### 2.8. Evaluation of prebiotic potential of CFP

#### 2.8.1 Growth behavior of probiotic strains in synthetic culture media containing CFP

**Fig. 5:**
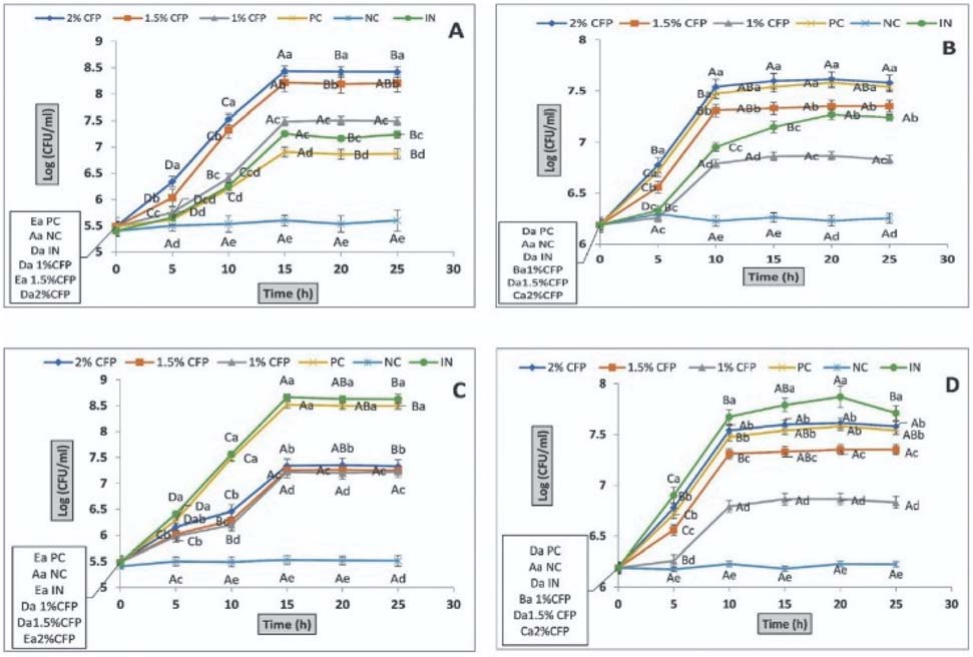
The effect of different concentrations of CFP on the growth behavior of *L. casei* (A), *L. rhaminosus* (B), *E. coli* Nissle 1917 (C) and *E. faecalis* (D). Positive control (PC), negative control (NC) and inulin (IN). Different letters (upper cases) were significant (*P* < 0.01) *vs.* time and different letters (lower cases) were significant (*P* < 0.01) *vs.* concentrations of CFP.

The growth of four probiotic strains (*L. casei*, *L. rhamnosus*, *E. coli* Nissle 1917 (EcN), *E. faecalis*) were compared using culture media supplemented with different concentrations of CFP (Fig. 5). All strains grew in culture media enriched with different CFP concentrations (1, 1.5 and 2%). The growth rate and final growth were directly proportional with CFP concentration. The final growth and growth rate of *L. casei* in culture media containing different CFP concentrations was significantly higher compared with the positive control (lactose) or standard prebiotic (inulin) (*P* < 0.01). In addition, faster growth at higher CFP concentrations (2%) was observed compared with other concentrations (Fig. 5A). The results indicated that there was a significantly (*P* < 0.01) progressive increase in the viability of *L. casei* and EcN during the incubation period from zero to 15 h (Fig. 5A and C). After 15 h, the viability of *L. casei* and EcN significantly (*P* < 0.01) decreased (Fig. 5A and C). In addition, there was no significant difference (*P* < 0.01) in the viability of *L. casei* or EcN between the incubation times of 20 or 25 h at all concentrations of CFP (Fig. 5A and C). Both *L. Rhamnosus* and *E. faecalis* exhibited significant differences (*P* < 0.01) in growth rate and final growth between 2% CFP and the other concentrations, whereas there was no significant difference (*P* < 0.01) in viability of both *L. rhamnosus* and *E. faecalis* between positive control and 2% CFP (Fig. 5B and D). There was no significant difference (*P* < 0.01) in *L. rhamnosus* and *E. faecalis* viability after 15, 20, or 25 h of incubation time (Fig. 5B and D). The growth rate and the final growth of EcN with lactose (positive control) were significantly (*P* < 0.01) higher compared with all CFP concentrations. Moreover, there were significant differences (*P* < 0.01) between the viability of EcN grown in culture medium enriched with 2% CFP compared with the other concentrations (Fig. 5C).

The final growth and growth rate of *E. faecalis* in culture media containing 2% inulin (standard prebiotic) was significantly higher compared with the different concentrations of CFP and positive control (lactose) (Fig. 5D), as well as the growth rate of EcN in culture media containing 2% inulin was significantly higher than different concentrations of CFP, whereas there was no significant difference (*P* < 0.01) in viability of EcN between inulin (standard prebiotic) and lactose (positive control) (Fig. 5C). The results indicated that there was no a significantly (*P* < 0.01) progressive increase in the viability of all probiotic strain in negative control during the incubation period (Fig. 5A–D).

The data regarding the growth of probiotic strains revealed that CFP at different concentrations (1 to 2%) as a carbon source may be considered suitable for growing probiotic strains, whereas the rates of growth of these strains were different based on CFP concentration. Milk sugar (lactose) was used as a positive control since it is the main sugar in milk and dairy products. *Enterococci*, *lactobacilli*, and *E. coli* Nissle 1917 are acclimated to this sugar. Despite the medical and pharmaceutical benefits of *Cassia fistula*, previous studies have not described the use of CFP as a prebiotic. All strains under study fermented CFP at different rates. Therefore, CFP metabolism may be varied in each strain. This could explain why *L. casei* has a high capacity to utilize CFP because it contains a high percentage of glucose compared with other monosaccharides. These results are consistent with those of^47^ who reported that *L. casei Shirota*, *L. casei*, and *L. johnsonii* are grouped in the same cluster according to fermented gluco-oligosaccharides (GOS). However, *L. rhamnosus* GG is classified in various clusters because of its ability to utilize fructo-oligosaccharides (FOS).

#### 2.8.2 Prebiotic index (PI) of CFP

**Fig. 6:**
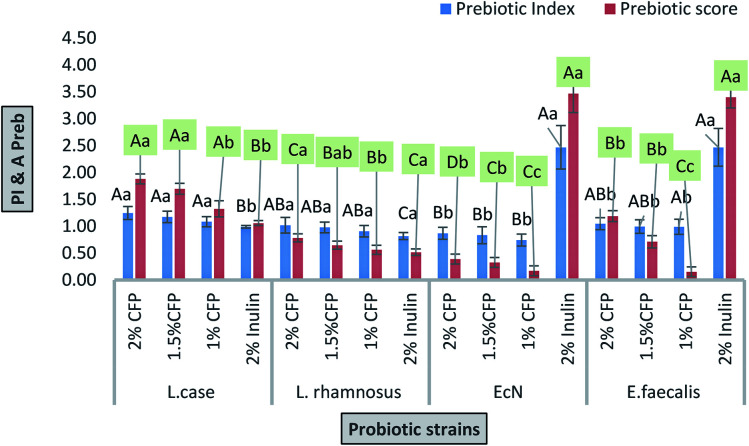
Prebiotic index and prebiotic scores of probiotic strains grown in different concentrations of CFP and inulin (standard prebiotic). Different letters (upper cases) were significant (*P* < 0.01) *vs.* strains and different letters (lower cases) were significant (*P* < 0.01) *vs.* concentrations of CFP in each strain.

The prebiotic index values presented in Fig. 6 were derived from the growth behavior of these probiotic strains according to eqn (1). The highest PI values were observed for EcN and *E. faecalis* incubated with 2% inulin (standard prebiotic) compare to different concentrations of CFP (Fig. 6). There was a significant difference (*P* < 0.01) between the PIs of *L. casei* and EcN at different CFP concentrations, whereas there were no significant differences (*P* < 0.01) between the PIs of *L. casei*, *L. rhamnosus*, and *E. faecalis* (Fig. 6). The PI values were observed for *L. casei* incubated with 2%, 1.5%, and 1% CFP (1.25 ± 0.12, 1.17 ± 0.11, and 1.08 ± 0.10, respectively), followed by *E. faecalis* and *L. rhamnosus* grown in 2% CFP (1.05 ± 0.11 and 1.02 ± 0.14, respectively). However, the lowest values (*P* < 0.01) were observed for EcN grown in 1, 1.5, and 2% CFP (0.74 ± 0.11, 0.83 ± 0.15 and 0.87 ± 0.10, respectively) followed by *L. rhamnosus* grown in 1 and 1.5% CFP (0.91 ± 0.10 and 0.98 ± 0.10). Also, *E. faecalis* exhibited a prebiotic index below one when grown on 1% CFP (0.99 ± 0.14) (Fig. 6). There were no significant differences (*P* < 0.01) among the different CFP concentrations for each strain (Fig. 6). A low prebiotic index value was obtained if the probiotic strain showed reduced growth on the tested sugar compared with the control (lactose).

The effects of prebiotics reported in previous studies are expressed in a qualitative way. PI is considered a quantitative value for comparing various polysaccharides. If the value resulting from eqn (1) is near or higher than 1, it indicates that probiotic growth is activated by polysaccharide compared with the control (lactose).^48^ All PI values for CFP (2%) obtained in the present study were significant (*P* < 0.01), except for *E. coli* Nissle 1917, where none of the CFP concentrations induced growth compared with the positive control (lactose).

#### 2.8.3 Prebiotic activity score (*A*_preb_) of CFP

The *A*_preb_ values presented in Fig. 6 were derived from the growth behavior of these probiotic strains according to eqn (2).^49^ There were significant differences (*P* < 0.01) between the *A*_preb_ for each of the four strains at each CFP concentration, except for EcN and *E. faecalis* at 1% CFP, and *L. rhamnosus* and *E. faecalis* at 1.5% CFP (Fig. 6). The highest *A*_preb_ values were observed for EcN and *E. faecalis* incubated with 2% inulin (standard prebiotic) compare to different concentration of CFP (Fig. 6). The *A*_preb_ values were obtained for *L. casei* incubated with 2%, 1.5%, and 1% CFP (1.88 ± 0.10, 1.70 ± 0.10 and 1.33 ± 0.15, respectively), followed by *E. faecalis* grown in 2% CFP (1.19 ± 0.10). The lowest values were observed for *E. faecalis* and EcN gown in 1% CFP (0.15 ± 0.10 and 0.17 ± 0.10, respectively) followed by EcN grown in 1.5 and 2% CFP (0.33 ± 0.10 and 0.39 ± 0.09, respectively). There were significant differences (*P* < 0.01) between *A*_preb_ at 2% and 1% CFP for each strain (Fig. 6). All values of *A*_preb_ were positive which indicated that the growth rate of the probiotic strains in prebiotic were higher compared with the enteric bacteria (*E. coli* K12) using prebiotic. A negative value for *A*_preb_ resulted if the examined probiotic strains grown in the tested polysaccharide was less than enteric bacteria growth on the tested polysaccharide.^27^

The prebiotic rule requires specific activation of probiotic bacteria coinciding with the inhibition of pathogenic bacterial growth. In this work, different concentrations of CFP induced the growth of enteric bacteria (*E. coli* K12) rather than inhibiting growth. However, the rate of growth of the probiotic was higher compared with enteric bacteria (*E. coli* K12) after 24 h of fermentation time.

The inquiry as to whether prebiotics are selective agents towards probiotic strains has been discussed and there are other studies that present prebiotics use within pathogenic bacteria.^2,50,51^ The ability of pathogenic bacteria to use prebiotic as a carbon source *in vivo* is possible according to *in vitro* studies. The prebiotic effect was factually indirect because of their ability to change the composition of gastrointestinal microbiota.^2^ Moreover, various individuals harbor various species of bacteria and there are many factors such as disease, diet, age, and drugs that affect the microbiota composition.^52^

#### 2.8.4 Determination of hydrolysis potential of CFP by using α-amylase

**Fig. 7:**
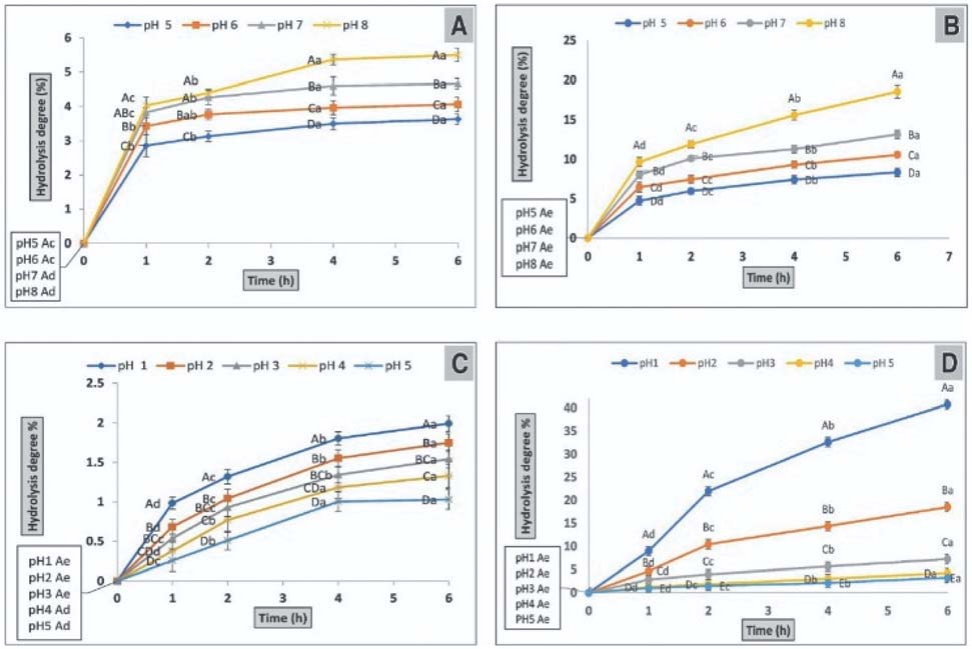
Determination of the hydrolysis potential of CFP (A) or inulin (B) by α-amylase at different pH levels. The percentage of hydrolysis potential of CFP (C) or inulin (D) by using AGJ at different pH levels different letters (upper cases) at the same time and different letters (lower cases) at the same pH were significant, at least at (*P* < 0.01).

The degree of hydrolysis CFP and inulin by α-amylase was determined at four pH values (5, 6, 7 and 8) and after incubation at 37 °C for different times (1, 2, 4 and 6 h) (Fig. 7A and B). The hydrolysis degree of CFP and inulin by α-amylase is directly proportional to progress of incubation period and also with the raise in pH (Fig. 7A and B). The hydrolysis of CFP by α-amylase was significantly (*P* > 0.01) increased and associated with incubation time from zero to 4 h at different pH levels, but there were no significant (*P* > 0.01) differences at 4 to 6 h incubation times (Fig. 7A). However the hydrolysis of inulin by α-amylase was significantly (*P* > 0.01) increased and associated with incubation time from zero to 6 h at different pH levels. The hydrolysis resistance rates for CFP (96.37% at pH 5, 95.93% at pH 6, 95.33% at pH 7, and 94.50% at pH 8) after 6 h were significantly (*P* > 0.01) decreased when the pH was increased from 5 to 8 (Fig. 7A). However inulin presented lower 91.66%, 89.44%, 86.86%, and 81.44% of hydrolysis resistance at pH 5, 6, 7, and 8, respectively, after 6 h (Fig. 7B). Therefore, higher resistance of CFP against α-amylase than inulin presented its suitability as prebiotic. The presence of the β-linkage between glucose in CFP makes it more resistant against to cleavage by α-amylase. The hydrolysis resistance of CFP was closed to FOS (∼95%).^53^ Crude polysaccharides that exhibit resistance to hydrolysis in the upper part of the gastrointestinal tract are considered as prebiotic.^54^ The degree of hydrolysis increased by prolonging the incubation time from 1 to 4 h and remained steady from 4 to 6 h. The similar resistance of CFP against α-amylase compared with FOS may result from its structural properties. The monosaccharide units in CFP are joined together by β-glycosidic bonds which were identified in the IR analysis. Our results are consistent with that of^55^ who reported that FOS is joined by 1,2-glycosidic bonds and possesses more hydrolysis resistance against human enzymes.

#### 2.8.5 Determination of the hydrolysis potential of CFP using AGJ

The ability of CFP to resist hydrolysis in an acidic environment, which is considered one of critical features for determining prebiotic potential in addition to its utilization by colonic probiotics.^22^ The hydrolysis of CFP and inulin by AGJ was determined at five pH levels (1, 2, 3, 4, and 5) and after incubation at 37 °C for different times (1, 2, 4, and 6 h) as shown in (Fig. 7C and D). A decrease in hydrolysis of CFP and inulin was significantly (*P* > 0.01) associated with increased pH ranging from 1 to 5 using AGJ (Fig. 7C and D). The hydrolysis of CFP by AGJ significantly (*P* > 0.01) increased with incubation time from 0 to 6 h at pH 1, 2, and 3, but there were no significant (*P* > 0.01) differences at 4 to 6 h incubation times at pH 4 or 5. However, the hydrolysis of inulin by AGJ significantly (*P* > 0.01) increased with incubation time from 0 to 6 h at five pH levels. The hydrolysis resistance rates for CFP (98% at pH 1, 98.25% at pH 2, 98.46% at pH 3, 98.67 at pH 4, and 98.97% at pH 5) after 6 h were significantly (*P* > 0.01) increased when the pH was increased from 1 to 5 (Fig. 7C). However, inulin presented lower 59.23%, 81.47%, 92.77%, 95.76%, and 96.73% of hydrolysis resistance at pH 1, 2, 3, 4 and 5, respectively, after 6 h (Fig. 7D). CFP presented 20.49, 10.59, 4.69, 3.18, and 3.17 fold higher resistance at pH 1, 2, 3, 4 and 5, respectively to hydrolysis against AGJ compare with inulin (Fig. 7C and D). This results presented that CFP will be available in the small bowel, where it presented more resistance to hydrolysis at low pH of AGJ from stomach. In addition to CFP resistance to acidic environment makes it beneficial for acidic foods such as fermented milk as also reported earlier.^56^ The main cause of partial CFP hydrolysis was probably because the glycosidic linkage was more readily broken at low pH values. The high resistance of CFP against acid condition reflects the possibility that it contains a high percentage of GOS.^53,57^ Several factors including linkages, rings, and monosaccharide composition may influence the hydrolysis of polysaccharides when subjected to AGJ as described.^58^

### 2.9. Influences of CFP on NO production in macrophages

**Fig. 8:**
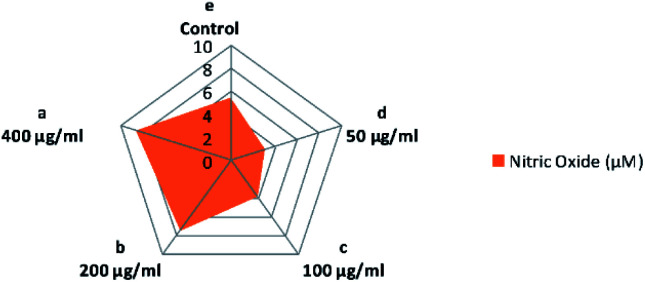
Influence of various concentrations of CFP on the production of NO in RAW 264.7 macrophages. Different letters (lower cases) are significant at *P* < 0.01.

It is known that nitric oxide plays an essential role in the immune system. It is defined as an essential product produced by macrophages activated by microbial compounds or cytokines.^59^ Griess reagent can be utilized to indirectly measure NO synthesis by determining the total nitrate concentration in a mixture of polysaccharide-activated macrophages. In this study, CFP induced a two-way adjusting influence on the production of NO by macrophages (Fig. 8). Incubation at low concentrations of CFP (0.05–0.1 mg mL^−1^) with macrophage cells resulted in a significant (*P* > 0.01) decrease in NO production compared with control cells (Fig. 8). However, higher CFP concentrations (0.2–0.4 mg mL^−1^) significantly induced NO production (*P* < 0.01). Previous studies showed that NO is an inflammatory mediator that stimulates macrophages with cytotoxic or cytostatic activity against bacteria, viruses, protozoa, fungi, tumor cells, and helminths. However, high concentrations of NO causes damage to different normal host cells and represses the proliferation of lymphocytes.^60^ The effects of two-way adjusting on NO synthesis exhibited by CFP indicates an application in suppressing NO production and macrophage activation.^60,61^

### 2.10. The effect of CFP on the bacterial fermentation time of milk

**Fig. 9:**
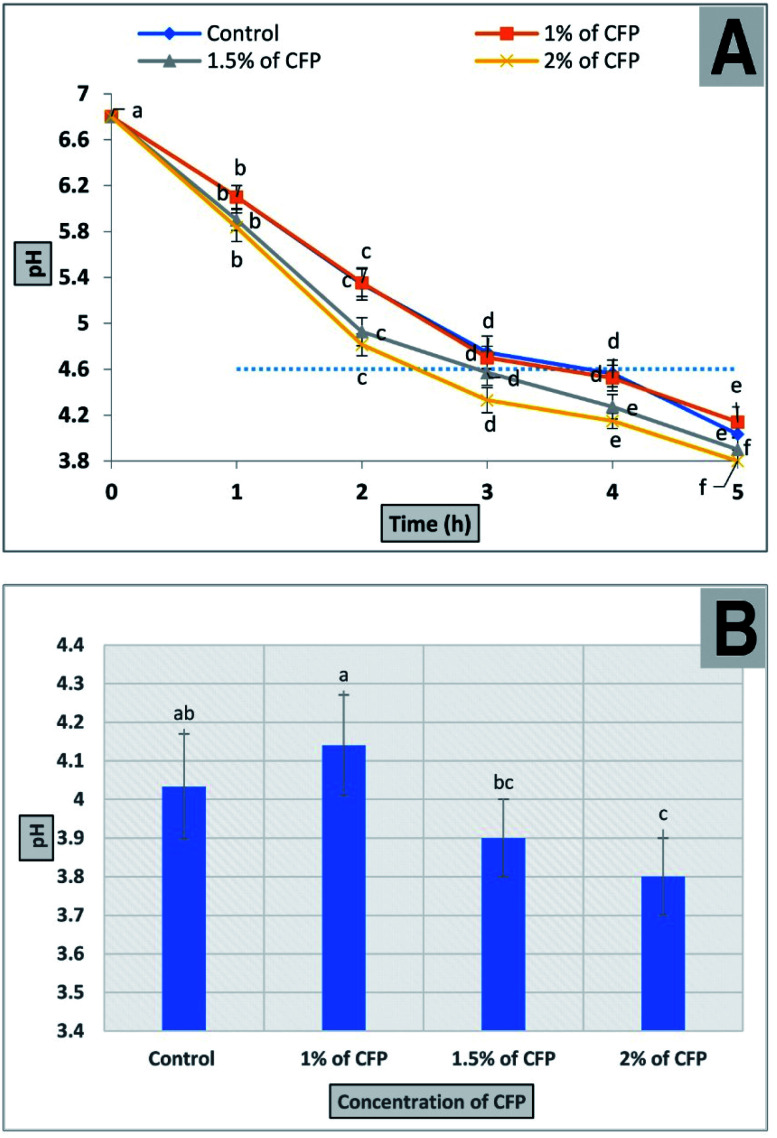
The effect of different CFP concentrations on milk acidity (A) a progressive decline in the pH of milk samples containing different concentrations of CFP inoculated with yogurt starter culture and incubated for 5 h. (B) The final pH of different treatments after 5 h. All points in the figure are labeled to present significant differences between different CFP concentrations within different letters (lower cases). Vertical bars present the standard deviation between the treatments mean.

The production of fermented milk (such as yogurt) is complete when the pH value reaches 4.6 ± 0.1 and is associated with full coagulation of the milk protein. The results for the bacterial fermentation time of milk containing different concentrations of CFP (1, 1.5, and 2%) are shown in Fig. 9. The time required to reach the desired pH or for full coagulation of fermented milk improved significantly (*P* < 0.01) by adding 1.5 or 2% CFP. On the other hand, there was no significant difference between 1% CFP and the control (Fig. 9). Using 1.5 and 2% CFP for cultured milk production significantly shortened (*P* < 0.01) the total fermentation time required to reach pH 4.6 (isoelectric point of milk) from 210 min to 180 and 150 min, respectively (Fig. 9A). The pH of the fermented milk sample enriched with 2% CFP after 5 h significantly (*P* < 0.01) decreased compared with the sample containing 1% CFP or the control, but there were no significant (*P* < 0.01) differences between 1.5 or 2% CFP (Fig. 9B). The bacterial fermentation time of milk was inversely proportional with the concentration of CFP (Fig. 9A). Consistent with our findings, the yogurt fermentation time was reduced by up to 10% was linked to supplementation with inulin.^62^ This corroborates the effect of prebiotic reported by other researchers for both lactobacilli^63^ and bifidobacterial.^63^ The reduction in the fermentation time of milk at different CFP concentrations in this study was consistent with the work of^34^ who confirmed that the addition of 1 or 2% ulvan polysaccharide significantly decreased the incubation time in the manufacture of synbiotic yogurt. Moreover, the reduction in incubation time of yogurt by adding CFP decreased the hardness of the yogurt, where a fast acidification rate depletes colloidal calcium phosphates from casein micelles, which results in individual casein release from micelles, followed by the formation of the casein network.^64^ The hardness of yogurt containing CFP decreased thus creating a soft and smooth yogurt curd which covers the mouth during mastication.^65,66^ The above results indicated the possibility of using CFP in the production of synbiotic yogurt with favorable physical and chemical characteristics.

## 3. Experimental

### 3.1. Materials

Mature fruits from the golden shower tree (*Cassia fistula*) were obtained from the campus of Mansoura University, Mansoura, Egypt in August 2019. The mature fruits were collected from a medium-sized tree that was approximately 15.6 m tall. The *Cassia fistula* fruit is a legume measuring 1.5–2.5 cm in width and 30–60 cm in length with a pungent flavor, a dark brown color, and containing many seeds. The mature fruits used in this study were randomly sampled in four directions from each of a total of ten trees. Two or four mature fruits within every direction were gathered and the mature fruits were combined for the subsequent steps in the study.

Absolute ethanol and anhydrous sodium acetate were obtained from Merck (Germany). Methanol and sulfuric acid were purchased from the Sigma chemical Co. (St. Louis, MO, USA). Standard monosaccharides (glucose, fucose, fructose, galactose, mannose, arabinose, and rhamnose) were obtained from the Shanghai Aladdin Chemical Reagent Company (Shanghai, China). All other reagents were of analytical grade. Stock solutions of the previously mentioned monosaccharides were prepared by dissolving them in deionized water (1000 mg L^−1^), storing at 4 °C, and diluting immediately before use.

All bacterial strains (*Lactobacillus casei* MSD102, *Lactobacillus rhamnosus* ML57, *Escherichia coli* Nissle 1917, *Escherichia coli* K12 and *Enterococcus faecalis* OD21) were used in this study. They were achieved from stock strains collection of microbiology laboratory (Dairy department, Faculty of Agriculture, Mansoura university, Egypt). Bacterial culture media (de Man, Rogosa and Sharpe (MRS) agar and Nutrient agar) were purchased from Thermo Fisher Scientific (Cairo, Egypt).

Murine macrophage cell line (RAW 264.7) were obtained from American Type Tissue Culture Collection (ATCC, USA). Cell culture reagents and tissue culture media were purchased from Thermo Fisher Scientific (Cairo, Egypt).

### 3.2. Samples preparation

#### 3.2.1 Extraction procedure

The peels of the mature fruits were carefully removed by hand and discarded. The pulp containing seeds were manually dissociated and the seeds were discarded. The sticky pulps of *C. fistula* were combined and air-dried for 72 h. The dried pulp (4.5% moisture) was then macerated overnight with petroleum ether to remove hydrocarbons and other neutral lipids. The resultant residue (1000 g) was extracted for 3 h with deionized water (1 : 12, g mL^−1^) in a water bath at 70 °C. The water-insoluble material was then removed by filtration and the material was re-extracted 3 times using the same procedure. The combined aqueous extract was then concentrated under low pressure at 55 °C. Precipitation was performed by gently adding 75% (v/v) ethanol to the solution and the resulting mixture was stored at 4 °C for 24 h. The mixture was then centrifuged (12 000 rpm, 15 min) to obtain a crude polysaccharide precipitate.

#### 3.2.2 Determination of total carbohydrate content

The total carbohydrate content of CFP was determined by the classical phenol–sulfuric acid method as described by ref. 67.

#### 3.2.3 Determination of reducing sugars

The reducing sugar content was determined spectrophotometrically according to the method described by ref. 68. Total polysaccharide content was calculated by subtracting the reducing sugar content from the total carbohydrate content.

#### 3.2.4 Estimation of protein content

Nitrogen content was determined according to ref. 69 and crude protein was calculated using the resulting factor.

#### 3.2.5 Monosaccharide profile analysis

The crude polysaccharide component of *C. fistula* (2.0 mg) was treated with 4 mL of 2 M H_2_SO_4_ at 105 °C for 150 minutes. The resulting liquid was diluted to a volume of 10 mL. A chromatographic technique was done using Dx-120 ion chromatography (Dionex, Sunnyvale, CA, USA) according to the method described by ref. 17.

#### 3.2.6 Molecular weight determination

A high performance liquid chromatography (HPLC) system (2695) supplied with a Waters Alliance 2414 RID A refractive index detector and a TSK-Gel G-3000-SW column (7.5 mm × 300 mm) was used to estimate the MW of the CFP samples. The column temperature and the detector were set to 30 °C. The concentration of CFP was 1 g L^−1^ and the injection volume was 50 μL. The eluant was distilled water which was delivered through a Millipore filter (0.45 μm) and the flow rate was 1 mL min^−1^. Calibration of the column was done by reference to a series of dextrans.

### 3.3. Infrared (IR) analysis

The crude polysaccharide was analyzed using the FTIR spectrum. A mixture of CFP was ground with dry KBr and pressed into a mold. The IR spectrum was collected in the 4000–400 cm^−1^ region using a Bruker Vectore 22 Fourier transform infrared spectrophotometer.

### 3.4. Amino acids composition

The amino acid composition of CFP was evaluated using a Hitachi L-8900 automatic amino acid analyzer (AAA). Acid hydrolysis was done by mixing 5 mg of CFP with 6 N HCl in sealed tubes at 105 °C for 18 h under vacuum. The resulting solution was evaporated and the nascent residue was re-dissolved in 0.02 M HCl followed by passage through a 0.45 μm nylon filter prior to injection into the AAA.^70^

### 3.5. Analysis of carbohydrate–peptide linkage

The β-elimination reaction was used to examine the O-glycosidic linkage in CFP. Briefly, 2.0 mg of CFP were dissolved in 10 mL 0.2 M NaOH solution and the reaction was carried out at 55 °C for 6 h.^71^ The solution including basified and non-basified CFP was monitored from 200 to 400 nm using a UV-1800 spectrophotometer. The N-glycosidic linkage was permanently kept stable in basic media whereas the O-glucosidic linkage was readily broken.

### 3.6. ^1^H-NMR of the *C. fistula* polysaccharide

The CPF sample (15 mg) was dissolved in deuterated methanol (CD_3_OD) and the ^1^H NMR was measured on Bruker Avance III spectrometer with solvent peaks as an internal reference. The ^1^H spectrum was recorded with fid resolution 0.12, spectrometer frequency 400.20 MHz, temperature 293.1 K, and line broadening 0.30 Hz.

### 3.7. Evaluation of prebiotic potential of CFP

#### 3.7.1 Effect of CFP on the growth of probiotic strains

The influence of different concentrations of CFP on probiotic strains was determined according to the method described by ref. 49. The assay was carried out by adding 10^6^ CFU of an overnight culture of probiotic strains (*L. casei* MSD102, *L. rhamnosus* ML57, and *E. faecalis* OD21) to separate tubes including MRS (without carbohydrates) broth. However the nutrient broth (NB) used for *E. coli* Nissle 1917 (EcN). Both culture media (MRS broth and NB) were supplemented with different concentrations (1, 1.5, and 2%) of CFP and 2% lactose was used as a positive control. The inulin (2%) has been used as standard prebiotic. The MRS broth and NB without sugar were used as negative control. The rate of the growth was determined each 5 h, by counting number of colony-forming units (CFU) within MRS agar for lactic acid bacteria or Nutrient agar for *E. coli*, followed by 24 h incubation at 37 °C.

#### 3.7.2 Prebiotic index (PI) of CFP

The prebiotic index (PI) is the proportion of probiotic strain growth on culture media supplemented with different concentrations (1, 1.5, and 2%) of CFP or 2% of inulin was used as standard prebiotic to the growth of the probiotic strain in lactose. A PI greater than 1 indicates that the sugar has a beneficial effect on the growth of the probiotic strain. An PI near 1 indicates a low efficiency of the tested sugar. The PI was estimated according to eqn (1).^49^1
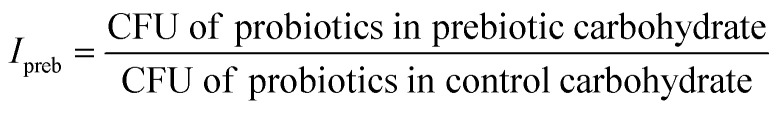


#### 3.7.3 Prebiotic activity score (*A*_preb_) of the CFP

The prebiotic activity score (*A*_preb_) was measured using mathematical eqn (2).2
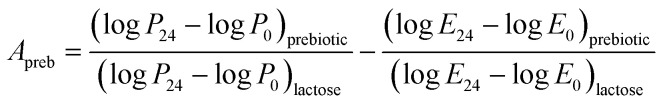


*A*_preb_ indicates the activity score of the prebiotic, log *P* is the probiotic bacteria growth (log CFU mL^−1^) at 24 h (*P*_24_) and 0 h (*P*_0_) on CFP (as numerator) and lactose (as a denominator). Log *E* indicates the *E. coli* K12 growth (log CFU mL^−1^) at 0 h (*E*_0_) and at 24 h (*E*_24_) of the strain on CFP (as a numerator) and lactose (as a denominator). Polysaccharides with high prebiotic activity scores activate prebiotic strain growth compared with growth on lactose. In contrast, a decrease of *E. coli* K12 growth may be observed compared with lactose.^49^ The inulin (2%) has been used as standard prebiotic.

#### 3.7.4 Evaluation of digestive potential of CFP using α-amylase

The influence of α-amylase on CFP or inulin (standard prebiotic) hydrolysis was determined by utilizing NaPO_4_ (20 mM) that was prepared at different pH values (5, 6, 7 and 8). CFP or inulin fraction was dissolved in this buffer to obtain 1% (w/v). Hydrolytic effects of each sample were estimated based on the method of ref. 53. Briefly, α-amylase solution was prepared by 6.7 mM NaCl (2 unit per mL; 5 mL) and mixed with equal volume of each tested sample. The mixture was incubated at 37 °C for 6 h. Samples obtained from each hour were tested to estimate the total and reducing sugars of the mixtures. Hydrolysis percentage of each tested sample was calculated based on eqn (3).

#### 3.7.5 Evaluation of the digestive potential of CFP using AGJ

The influence of AGJ on the hydrolysis of CFP or inulin (standard prebiotic) was assessed by calculating the degree of hydrolysis according to the method described by ref. 72. AGJ was prepared as follows: 14.35 g of Na_2_HPO_4_, 8.25 g of Na_2_HPO_4_. H_2_O, 0.2 g of KCl, 8 g of NaCl, 0.18 g of MgCl_2_·6H_2_O and 0.1 g of CaCl_2_·2H_2_O were dissolved in deionized water to fabricate a 1 L solution, followed by adjusting to five various pH values from 1 to 5 by using 5 M HCl. In detail, 50 mg of CFP or inulin fraction or dissolved in AGJ at different pH valued ranged between 1–5 and were adjusted to the final concentration to 1.0% (w/v), followed by incubation of samples at 37 °C for 6 h. The total sugar and reducing sugar of the mixtures were determined every 1 h. The hydrolysis degree of the tested samples was calculated according to eqn (3):3
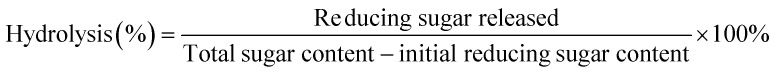


### 3.8. Effect of CFP on NO production in macrophages

RAW 264.7 cells (ATCC, USA) were suspended in Dulbecco's Modified Eagle's medium (DMEM) supplemented with 10% fetal bovine serum (FBS), 100 U mL^−1^ streptomycin, and 100 U mL^−1^ penicillin and treated with various concentrations of CFP (0.05, 0.1, 0.2, and 0.4 mg mL^−1^). The mixtures were then incubated at 37 °C, 5% CO_2_ for 24 h. A volume of 50 μL of Griess reagent was mixed with an equal volume of each supernatant in a new Eppendorf tube. After the mixture was maintained at room temperature for 15 min, the absorbance was recorded at 540 nm using an ELISA plate reader (Rayto RT-6000, Shenzhen, Egypt). The nitrite concentration was calculated by comparing the values with a standard curve of NaNO_2_ (0–100 μM). Cells that were incubated without CFP were considered a negative control.^60^

### 3.9. The effect of CFP on the bacterial fermentation time of milk

Milk fermentation was performed as described previously with slight modification.^73^ Briefly, reconstituted milk (10% w/v) enriched with 2% CFP was heated at 90 °C for 15 min followed by a cooling step at 43 °C. Yogurt starter culture (2%) was inoculated into the reconstituted milk. The milk samples were incubated at 43 °C followed by measurement of the changes in pH during fermentation. Then, 25 to 35 mL of each sample was poured in a 50 mL glass vial and the probe of pH meter was immersed into sample.

## 4. Conclusions

This study revealed that a new high molecular weight polysaccharide was extracted from *Cassia fistula* mature fruit pulp for the first time. This polysaccharide exhibited a low level of protein and aspartic acid was the predominant amino acid. Glucose and mannose were the predominant monosaccharides. The glycan–peptide linkage in the extracted polysaccharide structure was classified as an O-glycosidic type. Using CFP at different concentrations (1 to 2%) as a carbon source was considered suitable for growing various probiotic strains, whereas the growth rates of these strains were different based on CFP concentration. The final growth and growth rate of *L. casei* on culture media containing different CFP concentrations was significantly higher compared with the control (2% lactose) (*P* < 0.01). In addition, they grew faster at high CFP concentration (2%) compared with the other concentrations tested. The highest values for prebiotic index and prebiotic activity score were for *L. casei* at 2% CFP. In addition, the high resistance of CFP against α-amylase and acid conditions indicates that it may be considered as a prebiotic. The influence of two way adjusting on NO production induced by CFP indicated a possible application in the suppression of NO overproduction and activation of macrophages. The time required to reach the desired pH or for the full coagulation of fermented milk significantly decreased (*P* < 0.01) by adding 1.5% or 2% CFP, whereas there was no significant difference between 1% CFP and the control.

## Author contributions

All authors: conceptualization, methodology, software, data curation, formal analysis, visualization, writing – original draft, writing – review & editing.

## Conflicts of interest

There are no conflicts to declare.

## Supplementary Material
